# The Tardigrade Damage Suppressor Protein Modulates Transcription Factor and DNA Repair Genes in Human Cells Treated with Hydroxyl Radicals and UV-C

**DOI:** 10.3390/biology10100970

**Published:** 2021-09-27

**Authors:** Claudia Ricci, Giulia Riolo, Carlotta Marzocchi, Jlenia Brunetti, Alessandro Pini, Silvia Cantara

**Affiliations:** 1 Department of Medical, Surgical and Neurological Sciences, University of Siena, 53100 Siena, Italy; riolo@student.unisi.it (G.R.); carlottamarzocchi@libero.it (C.M.); cantara@unisi.it (S.C.); 2 Department of Medical Biotechnologies, University of Siena, 53100 Siena, Italy; jlenia.brunetti@unisi.it (J.B.); alessandro.pini@unisi.it (A.P.)

**Keywords:** Dsup, tardigrade, UV-C, oxidative stress, DNA repair, transcription factors

## Abstract

**Simple Summary:**

The Ramazzottius varieornatus is known to be the most resilient invertebrate on Earth. Belonging to the phylum of Tardigrada, it can live in any habitat, from the deep sea to various terrestrial environments, surviving in extreme temperatures, severe dryness or air deprivation. This exceptional tolerance to extreme conditions is attributable to the Dsup protein, which is able to bind and “protect” the DNA of this micro-animal, allowing it to survive where most other forms of life would quickly die. By introducing Dsup in human cell cultures, we investigated how this protein operates in response to two different extreme conditions: oxidative stress and ultraviolet (UV) irradiation. We learned that Dsup increases cell survival by triggering significantly different cellular mechanisms. In cells treated with hydrogen peroxide, Dsup “physically” protects DNA and activates several detoxification pathways aimed to remove intracellular free radicals. In contrast to this, a direct protection of DNA is not exerted by Dsup after UV irradiation, but the protein seems to activate mechanisms of DNA damage repair more efficiently, promoting faster cell recovery and survival. Even though further studies are required, understanding the mechanisms associated with Dsup resistance to cell damage may represent an important benefit for humans and plants.

**Abstract:**

The *Ramazzottius varieornatus* tardigrade is an extremotolerant terrestrial invertebrate with a length of 0.1–1.0 mm. These small animals show an extraordinary tolerance to extreme conditions such as high pressure, irradiation, chemicals and dehydration. These abilities are linked to a recently discovered damage suppressor protein (Dsup). Dsup is a nucleosome-binding protein that avoids DNA damage after X-ray and oxidative stress exposure without impairing cell life in Dsup-transfected animal and plant cells. The exact “protective” role of this protein is still under study. In human cells, we confirmed that Dsup confers resistance to UV-C and H_2_O_2_ exposure compared to untransfected cells. A different transcription factor activation was also observed. In addition, a different expression of endogenous genes involved in apoptosis, cell survival and DNA repair was found in Dsup+ cells after H_2_O_2_ and UV-C. In UV-C exposed cells, Dsup efficiently upregulates DNA damage repair genes, while H_2_O_2_ treatment only marginally involves the activation of pathways responsible for DNA repair in Dsup+ cells. These data are in agreement with the idea of a direct protective effect of the protein on DNA after oxidative stress. In conclusion, our data may help to outline the different mechanisms by which the Dsup protein works in response to different insults.

## 1. Introduction

In 2016, the sequencing of the *Ramazzottius varieornatus* tardigrade genome ended with the discovery of a unique DNA-associated protein with the ability to protect DNA from irradiation stress [1]. This protein, termed the damage suppressor protein (Dsup), is hypothesized to be responsible for the extraordinary characteristics of tardigrades. Additionally known as water bears, tardigrades are small invertebrates (0.1–1.0 mm in length) that have adapted to living in numerous habitats, such as marine, freshwater and terrestrial environments [2]. In the absence of water, they can enter an anhydrobiotic state to survive harsh conditions. They can resist extreme temperatures [3,4], vacuum, high pressure [5], radiation [6], chemicals and direct exposure to open space [7]. How Dsup may exert its protective role is still a matter of study. Hashimoto et al. have shown that the protein directly interacts with free DNA [1]. Three years later, Chavez et al., in a purified biochemical system, proved that Dsup is a nucleosome-binding protein enriched (more than 60%) in serine, alanine, glycine and lysine (SAGK) residues [8]. SAGK are disorder-promoting amino acids [9] implied in the coverage of the chromatin and, consequently, DNA protection [8]. Similarly to its functions in vivo, Dsup-transfected human cells have higher resistance to X-ray [1] and oxidative stress [8]. Even tobacco plants benefit from Dsup transfection by acquiring protection against damaging stress such as genomutagens or radiation [10]. In plants, Dsup affects the expression of endogenous genes involved in DNA damage signaling and repair [10]. This opens the possibility that, in addition to a direct interaction and coverage of DNA, Dsup induces protection by altering the expression levels of endogenous genes critical for cell survival and proliferation. 

DNA damage may occur as a consequence of cellular metabolism or as a result of various exogenous factors. All these factors lead to damage to double-stranded DNA molecules, such as single-stranded DNA segments (ssDNA) or double-stranded DNA breaks (DSBs). Reactive oxygen species (ROS) usually cause the generation of DSBs, whereas exposure to non-ionizing UV radiation causes biochemical changes such as cyclobutane pyrimidine dimers (CPDs) and 6-4 photoproducts (6-4 PPs), which in turn lead to DNA structure alterations, formation of bends or curves [11,12], and the arrest of replication forks during replication [13].

Ataxia-telangiectasia-mutated (ATM) and ataxia-telangiectasia-mutated Rad-related (ATR) are the major regulators of the DNA damage response. Both ATM and ATR are large kinases phosphorylating Ser or Thr residues followed by Gln. DSBs lead to ATM activation, while ssDNAs mainly involve ATR. ATM activates its downstream kinase checkpoint, kinase 2, and works in coordination with the MRE11–RAD50–NBS1 (MRN) complex [14]. ATR activates downstream kinase Chk1 and Chk2, and works with TopBp1, Claspin and RAD9-RAD1-HUS1 (9-1-1) complex [15]. ATM exerts its action mainly at the G1, S and G2 checkpoints; ATR activation occurs during every S-phase of the cell cycle to repair damaged replication forks and to prevent the premature entry of cells into mitosis [16].

One of the critical upstream regulators of ATM and ATR is the BRCA1 DNA Repair Associated (BRCA1) protein. BRCA1 is one of the main factors in maintaining genome integrity in mammalian cells. It is also involved in repair mechanisms and checkpoint pathways together with the MRN complex [17].

To prevent ROS, cells can also activate a number of antioxidant enzymes. Catalase (CAT) is one of these, strongly mitigating oxidative stress by destroying cellular hydrogen peroxide to produce water and oxygen [18]. CAT actions are linked to superoxide dismutases (SODs). SODs dismutate superoxide anions to H_2_O_2_ that are catalyzed to H_2_O by CAT, peroxiredoxins (Prxs), or glutathione peroxidases (GPx) [19].

In this article, we investigated the role of the Dsup protein in ROS and UV-C protection in transfected human cells. We evaluated, for the first time, the activation of transcription factors in Dsup-transfected cells after these stresses and measured the expression of genes involved in DNA damage response and repair, cell survival and protection. We highlighted that Dsup acts by promoting specific transcription factor activation and by signaling pathways linked to DNA damage repair and cellular antioxidant activity.

## 2. Materials and Methods

### 2.1. Cell Transfection

The HEK293 cell line (mycoplasma-free, verified by N-GARDE Mycolpsma PCR reagent set, Euroclone) was kindly donated by Prof. Sandra Donnini (University of Siena). pCXN2KS-Dsup was a gift from Prof. Kunieda Takekazu (Addgene plasmid #90019; https://www.addgene.org/90019/ (accessed on 27 September 2021); RRID: Addgene_90019). Cells were maintained in Dulbecco’s modified Eagle’s Medium (DMEM) supplemented with 10% fetal bovine serum (FBS). The expression construct was transfected into HEK293 cells using Lipofectamine^®^ 2000 Reagent (Life Technologies), and stably transfected cells were selected by 700 µg/mL G418 (SERVA Electrophoresis GmbH) treatment for three weeks.

### 2.2. Evaluation of Dsup Transcript Presence by Endpoint and Real-Time Reverse Transcriptase-PCR

Total RNA was extracted from cell pellets using the SV Total RNA Isolation System (Promega) following manufacturer’s instructions, and reverse-transcribed using the M-MuLV-RH First Strand cDNA Synthesis Kit (Experteam). Dsup expression was evaluated by endpoint PCR using the following primers: forward 5′- TCCACAGAACCCTCTTCCAC-3′ and reverse 5′- GACGATGCCACATCCTTCAC-3′ (T annealing: 55 °C, 35 cycles, amplicon length: 560 bp). PCR products were visualized in a 2% agarose gel with ethidium bromide. Following 2, 5, 7 and 9 days after stabilized Dsup culture, expression was quantified with real-time RT-PCR using GAPDH as the reference gene and following the conditions reported by Hashimoto et al. [1].

### 2.3. Cell Viability

MTT metabolic assay (Vybrant^®^ MTT Cell Proliferation Assay Kit, Molecular Probes, Thermo Fisher Scientific Logo, Shanghai, China) was used to quantify cell viability. To evaluate Dsup-induced resistance against free radicals, both Dsup+ and untransfected HEK293 were seeded at 100,000 cells/mL density in a 96-well plate. After 24 h of incubation, cells were treated with 250, 500 and 1000 µM hydrogen peroxide (H_2_O_2_) for 4 h or O/N in complete medium (10% FBS, +/− G418). To evaluate Dsup-induced resistance against radiation, both Dsup+ and untransfected cells were plated at 100,000 cells/mL density in a 96-well plate. Before treatment, complete medium was removed and replaced with 100 µL PBS. Cells were exposed for 5″ or 15″ to UV-C (source 8 W lamp, 4 mJ/cm^2^). After treatment, cells were incubated in complete medium for 24 or 48 h before subsequent evaluations and then incubated at 37 °C for 4 h with the tetrazolium dye MTT (3-(4,5-Dimethylthiazol-2-yl)-2,5-Diphenyltetrazolium Bromide) (yellow) which, in healthy cells, is converted by mitochondrial enzymes into an insoluble formazan (purple). Solubilization was carried out with DMSO (50 µL) at 37 °C for 10 min. Then, the number of viable cells was determined by measuring absorbance at 540 nm in a micro plate reader (Tecan). Each experiment was run in triplicate and was repeated at least three times.

### 2.4. Cyclobutane Pyrimidine Dimers (CPDs) Evaluation

To measure DNA damage in terms of CPD formation, 100,000 Dsup+ and untransfected HEK293 cells/mL were plated in 6 cm diameter dishes and exposed for 5″ or 15″ to UV-C (source 8 W lamp, 4 mJ/cm^2^). DNA was extracted from three independent experiments, using QIAamp DNA micro kit (Qiagen) immediately after exposure (time 0) and after a recovery of 24 or 48 h. DNA was quantified by spectrophotometer analysis and for each sample the same amount of DNA was treated with T4 Endonuclease V enzyme (10 IU/µL) O/N at 37 °C. DNA fragments were run in a 1% agarose gel containing ethidium bromide at 90 V for 70 min [20].

### 2.5. Transcription Factor Evaluation

Evaluation of transcription factor activity was carried out by ELISA. Both Dsup+ and untransfected HEK293 were seeded at 100,000 cells/mL density in 6 cm diameter dishes. Cells were treated O/N with 250 and 1000 µM H_2_O_2_ or exposed to UV-C (source 8 W lamp, 4 mJ/cm^2^) for 5″ (recovery time 24 and 48 h). To obtain nuclear extracts, pellets were resuspended in 1 mL of cold buffer A (10 mM Hepes pH 7.9, 10 mM KCl, 0.1 mM EDTA, 0.1 mM EGTA, 1 mM DTT) added with 100 µL protease inhibitor cocktail (Sigma) and 500 mM PMSF, and incubated in ice for 15 min. Then, lysates were centrifuged for 2 min at 17,000 rcf (4 °C). Supernatants, containing cytoplasmatic proteins, were stored at −80 °C. Pellets were incubated in 50 µL cold complete buffer (provided by the kit) for 30 min at 4 °C. Then, samples were centrifuged for 5 min at 17,000 rcf (4 °C). Supernatants, containing nuclear extracts, were stored at −80 °C. Before assay, protein concentration was evaluated by Bradford (Sigma) and working aliquots of 1 mg/mL were prepared for each sample. Final tested concentration was 20 µg/mL. MAPK pathway (ATF2, p-c-Jun, c-Myc, MEF2, STAT1α) (Abcam), AP-1 family (c-Fos, FosB, Fra-1, p-c-Jun, JunB, JunD) and CREB/pCREB (Active Motif) were evaluated following manufacturer’s instructions. Each experimental point was run in triplicate.

### 2.6. Gene Expression Analysis

RT-qPCR was performed using Rotor-Gene Q (Qiagen) to analyze the expression of endogenous genes in Dsup+ and untransfected cells (100,000 cells/mL) after treatment, with 250 and 1000 µM of H_2_O_2_ for 4 h and O/N, and exposure to UV-C for 5″ or 15″ (recovery time 0, 24 and 48 h). RNA was extracted using SV Total RNA Isolation System (Promega) and reverse-transcribed using M-MuLV-RH First Strand cDNA Synthesis Kit (Experteam). FastStart Essential DNA Green Master Mix (Roche) was added to each tube together with 300 nM specific primers (available upon request). Annealing temperature was 60 °C. A melt curve was added at the end of each amplification to exclude the presence of non-specific products. Samples were normalized to GAPDH and ribosomal RNA 18 s and quantification was determined by using the 2^−ΔCT^ method. Each sample was run in triplicate and three biological replicates were performed. Sequences of the primers used for gene expression analysis are reported in Supplementary Table S1.

### 2.7. Statistical Analysis

GraphPad Prism software version 5 was used for statistical analyses. For qPCR analysis, at least three separate replicates for each assay were performed. Sample differences were assessed by one-way ANOVA test. Survival data and transcription factor activation data were analyzed using a paired test for non-parametric data (Wilcoxon signed-rank test). For all comparisons, a *p* value of <0.05 was considered significant.

## 3. Results

### 3.1. Cell Survival under Stress Conditions

**Figure 1:**
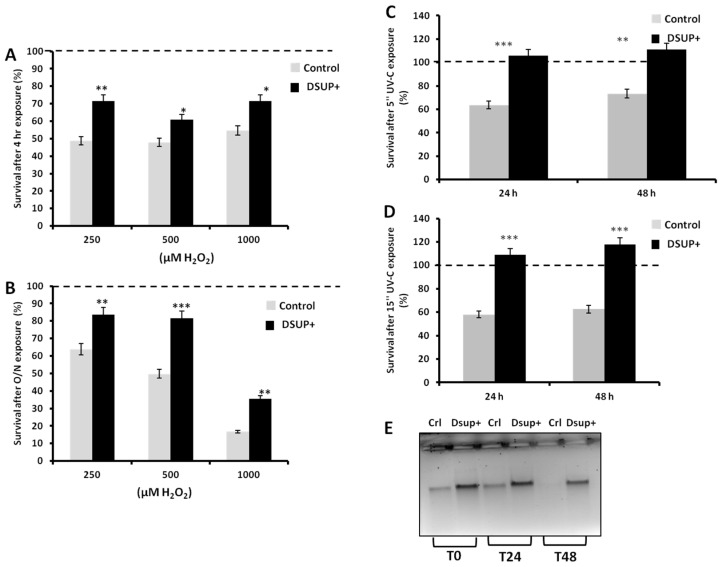
Cell survival after 4 h (**A**) or O/N (**B**) treatment with increasing (250, 500, and 1000 µM) concentration of H_2_O_2_. Survival after 5″ (**C**) or 15″ (**D**) of UV-C exposure and 24 or 48 h of recovery. In all figures, 100% represents the basal condition (dotted line), gray bars are untransfected cells (indicated as control), and black bars are Dsup+ cells. * *p* < 0.5; ** *p* < 0.01; *** *p* < 0.001 by Wilcoxon signed-rank test. Each experiment was run in triplicate and was repeated at least three times. (**E**) Representative gel out of three of cyclobutane pyrimidine dimers (CPDs) formation in Dsup+ and untransfected cells (indicated as control) exposed to 5″ of UV-C and treated with T4 endonuclease V enzyme. Please refer to the Full agarose gel in the supplementary.

To examine the effect of Dsup on cell death induced by Reactive Oxygen Species (ROS), we established a HEK293 cell line stably expressing Dsup protein (Dsup+) (Supplementary Figure S1A,B) and exposed it, together with untransfected HEK293, to 250, 500 or 1000 µM H_2_O_2_ for 4 h or O/N. In in vivo and in vitro models, these H_2_O_2_ dosages have been shown to induce apoptosis [21,22,23]. The percentage of cell survival (evaluated by MTT) was greater in Dsup+ cells for all treatments and at each time point (Figure 1A,B). At all concentrations, cell survival was lower than the basal condition (represented as 100% of survival, shown as a dotted line in figures).

Similarly, we exposed Dsup+ and untransfected cells to 5″ of ultraviolet light and observed a reduction in cell death and an increase in cell survival and growth (even above basal condition) in Dsup+ stable cells (Figure 1C) after 24 or 48 h of recovery. After 15″ of exposure, we obtained similar results with a comparable survival rate (Figure 1D). We chose an UV-C source, as UV-C is the shortest wavelength of the three forms of UV. The shorter the wavelength, the more harmful the UV radiation. The main target of UV-C irradiation in living cells is nuclear DNA. The formation of DNA lesions such as pyrimidine dimers inhibits DNA replication and causes chromosomal breakage and cell death. To evaluate whether cell protection mediated by Dsup was linked to a reduction in cyclobutane pyrimidine dimers (CPDs) formation, we exposed Dsup+ and untransfected HEK293 to 5″ or 15″ of UV-C and collected cell pellets for DNA extraction immediately or after 24 or 48 h of recovery. DNA was treated with T4 endonuclease V enzyme, which cleaves DNA at sites of CPD damage. As shown in Figure 1E, at all time points Dsup+ cells did not display CPD formation after 5″. Similar data were obtained after 15″ (not shown).

### 3.2. Transcription Factor Activation in Response to Stress Condition

**Figure 2:**
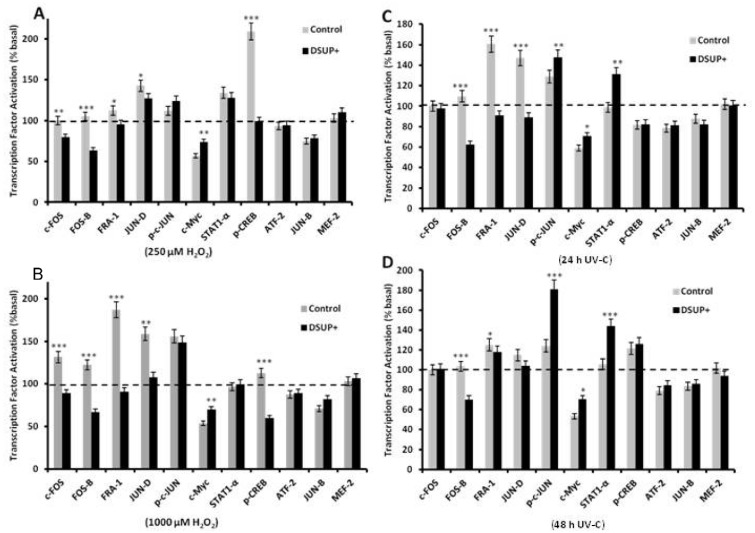
Transcription factor activation in Dsup+ and untransfected HEK293 (control) after 250 (panel (**A**)) or 1000 (panel (**B**)) µM H_2_O_2_ O/N or 5″ UV-C exposure (panel (**C**), 24 h of recovery and panel (**D**), 48 h of recovery). Results are reported as percentage over basal condition (100% and dotted lines in figure). MAPK pathway (ATF2, p-c-Jun, c-Myc, MEF2, STAT1α), AP-1 family (c-Fos, FosB, FRA-1, p-c-Jun, JunB, and JunD) and CREB/p-CREB expression were evaluated by ELISA. * *p* < 0.5; ** *p* < 0.01; *** *p* < 0.001 by Wilcoxon signed-rank test. Each point is the mean of triplicate wells.

To evaluate whether Dsup expression was able to modulate transcription factor (TF) activation, MAPK pathway (ATF2, p-c-Jun, c-Myc, MEF2, STAT1α), AP-1 family (c-Fos, FosB, Fra-1, p-c-Jun, JunB and JunD), and CREB/pCREB expression were assessed by ELISA. As shown in Figure 2, in untransfected cell treatment with 250 (panel A) or 1000 (panel B) µM H_2_O_2_ resulted in an up-regulation of c-Fos, FosB, Fra-1, JunD and p-CREB above basal condition (dotted line in figure). In Dsup+ cells, on the other hand, these TFs were down-regulated in comparison to the basal condition and were significantly inhibited compared to untransfected HEK293. Only c-Myc was down-regulated relative to control (dotted line) in both cell lines, but in this case we observed a significant up-regulation in Dsup+ cells compared to untransfected cells (Figure 2A,B). No variation was detected for p-c-Jun, STAT1α, ATF-2, JunB and MEF-2 in both cell lines and H_2_O_2_ concentrations.

Considering that outcomes on survival for 5″ or 15″ were comparable, we selected 5″ UV-C as time of exposure to be shown for transcription factor activation analysis. After irradiation, cells were allowed to recover for 24 h (Figure 2C) or 48 h (Figure 2D). Untransfected HEK293 showed a marked activation of Fra-1, JunD, and p-c-Jun, while no differences were observed for FosB and STAT1α. In Dsup+ cells, UV-C induced down-regulation of FosB, Fra-1, JunD, and in all cases a significant difference between untransfected cells and Dsup+ cells was observed. For p-c-Jun and STAT1α an up-regulation was evident in Dsup+ cells, and the p-c-Jun up-regulation was even significantly greater than that observed in untransfected HEK293. Only c-Myc was down-regulated relative to the control (dotted line) in both cell lines, and, again, we found a significant up-regulation in Dsup+ cells compared to untransfected cells (Figure 2C,D). No variation was detected for c-Fos, p-CREB, ATF-2, JunB and MEF-2 in both cell lines at 24 or 48 h.

### 3.3. Gene Expression in Response to Stress Conditions

**Figure 3:**
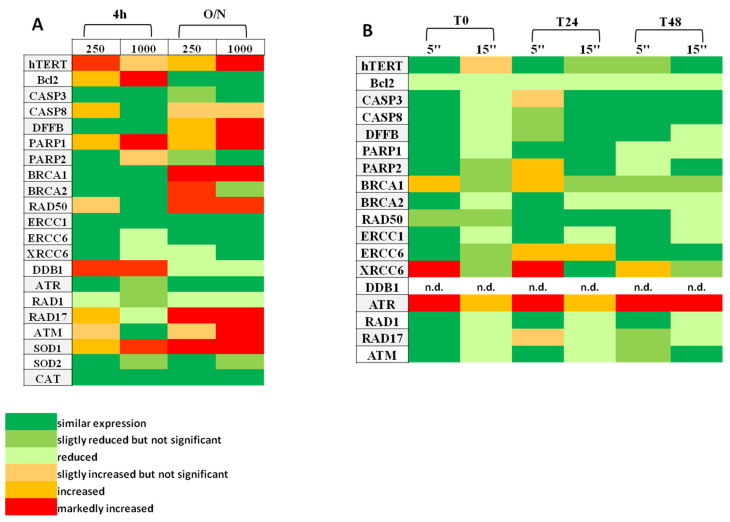
Changes in transcription levels of selected endogenous genes in Dsup+ cells compared to untransfected cells exposed to H_2_O_2_ (µM) (**A**) and UV-C (**B**) expressed as heatmap. n.d.: Not detected. Each sample was run in triplicate and three biological replicates were performed.

In order to explore whether Dsup affected the expression of endogenous genes under stress conditions (low to high dose H_2_O_2_ and UV-C exposure), RT-qPCR was performed to analyze genes involved in DNA damage response and repair, as well as in cell survival and protection from oxidative stress (Supplementary Table S2). As summarized in Figure 3A,B and in Supplementary Figures S2 and S3, for some of the analyzed genes, Dsup-expressing cells showed different transcription patterns from untransfected cells, which were used as the control. Specifically, Dsup+ cells treated with H_2_O_2_ (Figure 3A, Supplementary Figure S2) showed an increase in Bcl2 and, at some conditions, CASP8 genes with a reactivation of telomerase. In parallel, some of the genes involved in DNA repair and cell cycle checkpoint (PARP1, BRCA1-2, RAD50, RAD17, and ATM) were up-regulated, while others (ERCC6, XRCC6, and RAD1) were moderately down-regulated. DDB1 was up-regulated after 4 h of exposure but down-regulated at a longer time (O/N). SOD1 was markedly up-regulated in response to H_2_O_2_. In contrast, in Dsup+ cells exposed to UV-C (Figure 3B, Supplementary Figure S3) we observed an increase in some of the genes involved with DNA repair (XRCC6, ERCC6, ATR, and BRCA1), while others (BRCA2 and ERCC1) were down-regulated. A general hypoexpression was also observed for cell cycle checkpoint genes (RAD1, RAD17, and ATM).

**Figure 4:**
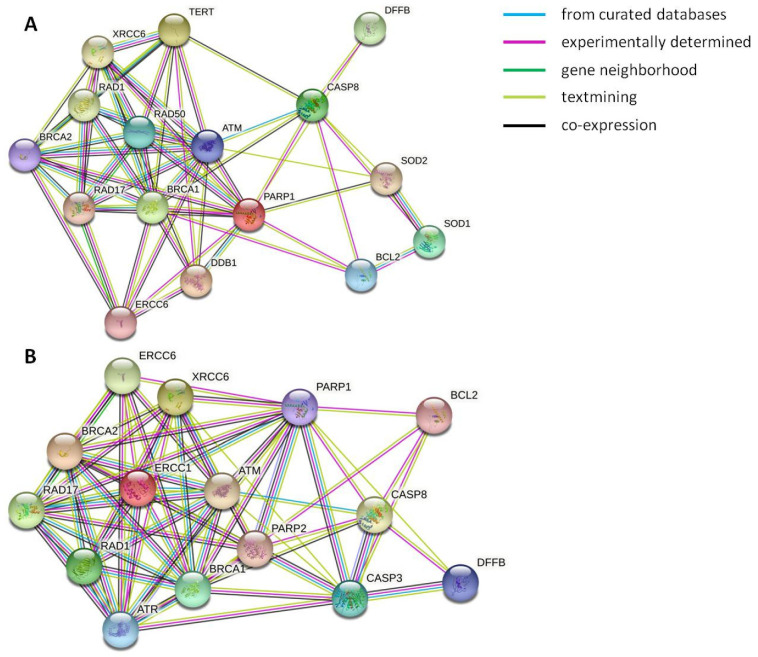
STRING protein–protein interaction network for differentially expressed genes after H_2_O_2_ treatment (**A**) and UV-C exposure (**B**). Colored lines between the proteins indicate the various types of interaction evidence: a green line indicates neighborhood evidence; a purple line indicates experimental evidence; a light green line indicates text-mining evidence; a light blue line indicates database evidence; a black line indicates co-expression evidence.

Results from expression studies showed that H_2_O_2_ and UV-C exposure involved different pathways to respond to cell damage. This phenomenon is depicted in Figure 4 in which String v.11.0 was used to evaluate protein interaction network of differentially expressed genes after H_2_O_2_ (Figure 4A) and UV-C (Figure 4B) treatments.

## 4. Discussion

In this study we investigated the cellular mechanisms responsible for the protection against external insults in mammalian cells expressing the Dsup protein. While Dsup expression has been shown to protect DNA from damages induced by H_2_O_2_ and improve mammalian cell survival, the scarce data available regarding UV-C response have been obtained only in plants. Furthermore, we are still at the very beginning of understanding the cellular pathway involved in Dsup-mediated protection from external insults. To this purpose, for the first time, to the best of our knowledge, transcription factor modulation and expression of gene pathways associated with damage response and repair were analyzed in Dsup+ and untransfected HEK293 cells exposed to oxidative stress and UV-C irradiation. In Dsup+ cells, we observed an increase in cellular survival in both oxidative stress and UV-C irradiation conditions, with a significant reduction of CDP formation in the case of UV-C exposure, in agreement with previous reported studies. However, the cellular mechanisms involved in the response to these insults appeared to differ significantly.

In the case of oxidative stress, untransfected cells responded to damage-activating pCREB and AP-1 family transcription factors (c-Fos, FosB, Fra-1, JunD). In the Dsup+ cells, the up-regulation of these factors was not observed. H_2_O_2_ increases the transcription of AP-1 so that when up-regulated it spontaneously concentrates in the nucleus to activate gene expression [24]. Similarly to AP-1, CREB activation is also induced by ROS and is critical for cell survival [25], promoting DNA repair after treatment with hydrogen peroxide. The activation of CREB in response to oxidative stress is dependent upon ATM kinase, which can either switch on or off CREB transcriptional activity, depending on the extent of DNA damage [26]. CREB signaling protects human neuronal cells from oxidative DNA damage not only by inducing anti-apoptotic genes and cell cycle arrest, but also by increasing the expression of genes responsible for DNA repair. In Dsup+ cells, CREB is down-regulated, suggesting that all these mechanisms are likely not activated. In addition, CREB is able to negatively regulate c-Myc and S phase induction [27]. This, in turn, could explain the up-regulation of c-Myc in Dsup+ cells. The induction of c-Myc may promote the G1/S phase transition and the cell cycle progression. On the other hand, c-Myc may play a role in determining cellular redox balance. It has been reported that c-Myc transcriptionally regulates g-glutamyl-cysteine synthetase (g-GCS), the rate-limiting enzyme catalyzing the biosynthesis of glutathione, the most abundant antioxidant and a major detoxification agent in cells [28].

At the gene expression level, in Dsup+ cells, among apoptotic genes, only Bcl2 expression increased after treatment with H_2_O_2_. Conversely, Dsup+ cells showed a constant increase over time of PARP1, hTERT, and SOD1 expression. PARP1 recruitment is one of the earliest events in DNA damage response following several types of insults, such as oxidative and metabolic stresses [29]. PARP1 recognizes DNA breaks and is involved in the early enrollment of factors that facilitate DSB repair [30], including BRCA1 [31], whose expression in our study was up-regulated in Dsup+ cells after O/N treatment with H_2_O_2_. hTERT is the catalytic subunit of the telomerase holoenzyme that, in addition to its role in the maintenance of telomeres, exhibits antioxidant activity. hTERT overexpression can decrease the basal cellular ROS levels but also inhibit endogenous ROS production [32]. Interestingly, it has been shown that c-Myc is able to stimulate hTERT expression, binding a c-Myc E-box within the hTERT proximal promoter [33]. Additionally, in this case, among the mechanisms by which hTERT seems to regulate the intracellular redox status, is the capability of modulating glutathione levels in the cells and conferring survival advantages. SOD1 is a well-characterized, ubiquitously-expressed and highly-conserved enzyme, considered a key regulator of antioxidant response. Overexpression and de novo synthesis of SOD1 mRNA has been reported in H_2_O_2_-treated cells [34]. SOD1 catalyzes the dismutation of a superoxide radical into an oxygen and hydrogen peroxide, which are further eliminated by other enzymes such as catalase [35,36]. Of note, the expression of ATM and ATR kinases, two key factors in DNA damage response caused by oxidative stress, did not display significant variations in Dsup+ cells. This suggests that their activity is not required, and Dsup+ cells can survive and proliferate without switching on the mechanisms of DNA repair. Taken together, all these data indicate that H_2_O_2_ treatment in Dsup+ cells only marginally involve the pathways responsible for DNA repair and suggest that the amount of DNA damage may be limited. This is in agreement with what was reported by Chavez and colleagues [8], who showed that Dsup is a nucleosome-binding protein able to preserve chromosomal DNA from hydroxyl radical-mediated cleavage and adds further information about the Dsup-mediated response in cells exposed to oxidative stress. Thus, it can be hypothesized that, while the DNA is “physically” protected from damages by Dsup, detoxification mechanisms aimed at removing ROS and limiting oxidative stress are activated (SOD1, hTERT, probably g-GCS), allowing cells to survive and grow. Interestingly, Dsup+ cell viability is higher after overnight treatment than 4 h treatment with H_2_O_2_, probably due to an increased induction of genes involved in the antioxidant response.

After UV-C exposure, the transcription factors (FosB, Fra-1, JunD, and p-c-JUN) were activated in untransfected cells, while Dsup+ cells exhibited an increase in p-c-Jun and STAT1α. In mammalian cells, STAT1α participates in type I interferon (IFN) pathways and activates type I IFN-response genes [37]. It has been shown that type I IFNs are key factors in normal skin growth and maintenance and are usually down-regulated in the majority of skin tumors [38]. UV exposure interferes with STAT1α activation, hindering IFN from exerting its protective effects [37,39]. It can, therefore, be hypothesized that STAT1α plays an important role in the response to UV damage. c-Myc acts in a similar way to p-c-Jun, a well-characterized regulator of the mammalian UV response, essential for the UV-irradiated cells to avoid growth stall and restart the cell cycle [40]. Interestingly, in our study c-Myc expression was stable after 24 h and 48 h and did not increase over time, as p-c-Jun and STAT1α. This may be related to the key role of c-Myc in cell cycle control. c-Myc can increase p53-dependent apoptosis, exerting a direct regulation on cell destiny after UV-C exposure and, acting together with p-c-Jun, can switch the response from cell cycle arrest recovery to apoptosis or vice-versa [41].

At gene expression level, we found a relevant up-regulation of ATR kinase expression, which is considered the main player in UV-induced DNA damage. ATR activation, occurring during the S phase, allows repairing stalled replication forks and avoiding the arrest of replication or premature entry into mitosis [16]. In addition, Dsup+ cells exhibited increased expression in other genes related to the ATR rescue pathway: BRCA1, which is recruited during the S/G2 phase and contributes to the UV irradiation response operating in gap repair at photoproduct-stalled replication forks level [42]; XRCC6, which participates in the UV-G2 checkpoint [43]; ERCC6, whose gene represents a potential target for inactivation by UV light and seems to act as a “dosimeter” of DNA damage (though DNA damage exceeds a certain threshold, ERCC6 transcript is depleted and cell death is promoted) [44]. In Dsup+ cells, ATM expression was similar or lower than that in untransfected cells. ATM activation occurs mainly under oxidative stress and double-stranded DNA breaks, while it is less involved in response to UV exposure [16]. In the same way, we observed a decrease in the expression of BRCA2, usually involved in response to DSBs [17]. Other factors related to UV-induced CPDs repair, such as RAD1 and RAD17, were down-regulated in Dsup+ cells. This may appear to be quite surprising; however, it is not unusual that the expression of gene responsible for UV damage response apparently remains unchanged or, rather, slightly decreases after UV exposure [45]. Additionally, in this case, a post-transcriptional regulation of these factors cannot be excluded.

**Figure 5:**
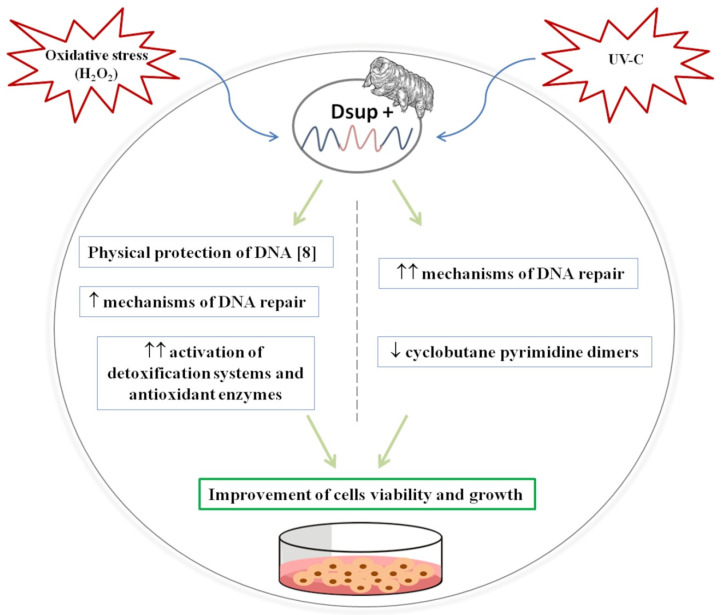
Schematic model of protection mechanisms in mammalian cells expressing Dsup protein (Dsup+) exposed to oxidative stress and UV-C irradiation. After H_2_O_2_ treatment, Dsup+ cells mainly activate the detoxification system and antioxidant enzymes limiting oxidative stress, while DNA repair mechanisms are moderately turned on, probably due to DNA “physical” protection mediated by Dsup [8]. On the other hand, after UV-C exposure, Dsup+ cells respond by activating more efficient DNA repair genes reducing cyclobutane pyrimidine dimers formation. All these protective mechanisms give Dsup+ cells a greater resistance to external stress, improving their viability and growth respect to untransfected cells. ↑: moderate; ↑↑: high; ↓: low/reduced.

All these data seem to suggest that, in contrast to what was observed for H_2_O_2_ treatment, DNA is not “physically” protected by Dsup after UV-C irradiation, but rather Dsup activates more efficient mechanisms of damage repair (STAT1, c-Myc, p-c-Jun; ATR, BCRA1). In this way, cells may remove CPDs and recover faster, mitigating the deleterious effects on cell survival (Figure 5).

The response to UV-C exposure of Dsup+ cells may seem a paradox. Usually, the extent of ATR activation reflects the degree of the damage load (in other terms, the more CPDs, the more ATR activation). In Dsup+ cells, instead, the amount of CPDs is lower. However, the expression of ATR is highly increased. These data are in agreement with those obtained in tobacco plants expressing the *Dsup* gene: transfected plants UV-C irradiated exhibited an increased survival, an enhanced ATR expression, and an improvement of DNA damage sensing and DNA repair pathways involving ATR [10]. On the other hand, those plants displayed fewer DSBs and generated less response from DSB signaling pathways than the plants without Dsup when exposed to genotoxic stresses. Therefore, all our results are consistent with those obtained in plants. However, this is the first time that such mechanisms are proposed in humans, and these findings suggest a common Dsup-related response in eukaryotic cells very different from each other.

We are aware that our study may have some controversial points. First of all, we have chosen to compare stable transfected Dsup+ cells to untransfected cells, rather than to empty-vector transfected cells. This has been a conscious decision, since it is known that stable transfection of an empty vector into HEK293 cells, as well as in other cell lines, is able to enhance chromosomal instability and genomic heterogeneity [46]. In addition, we have opted to perform our experiments using the same conditions as those described by Hashimoto and colleagues in the first study on the Dsup gene [1]. Furthermore, for the gene expression analysis, genes included in the study, although representative of key pathways, are only some of those potentially involved in DNA damage response and more extensive analysis (i.e., RNAseq) may be applied to check cellular transcriptome. However, RT-QPCR represents the gold standard for mRNA expression investigation. Finally, many of the corresponding proteins are regulated by phosphorylation/dephosphorylation, so a substantial uniformity in gene expression does not necessarily reflect uniformity in their activity. On the matter of gene expression, despite most of the expression profiles being different in H_2_O_2_ and UV-C, indicating a possible damage-dependent effect, we cannot exclude that Dsup expression per sè induces some sort of stress without any external source of damage partially affecting, like that, gene expression. Nevertheless, our data may help to delineate the different ways in which the Dsup protein operates in response to different insults. Further studies are needed to better characterize how Dsup works in the cells, and we believe that our study may lay the groundwork for a deeper understanding of its activity.

Conclusion: Our results delineate different mechanisms of action for the Dsup protein in response to different stimuli. Understanding such processes may be of great importance to generate plants more drought-tolerant or resilient to climate changes and desertification. In humans, the molecular biology of tardigrades with their amazing adaptation systems, may help cancer research on aspects such as genetic integrity, cell protection and DNA repair.

## Data Availability

Data is contained within the article or Supplementary Material.

## Supplementary Materials

The following are available online at https://www.mdpi.com/article/10.3390/biology10100970/s1. Figure S1: Confirmation of Dsup transfection evaluated by PCR on genomic DNA and expression, Figure S2: Relative expression of selected genes after H_2_O_2_ treatment, Figure S3: Relative expression of selected gene after UV-C treatment. Table S1: Primer sequences, Table S2: Gene ontology and gene actions.

## References

[1] Hashimoto T., Horikawa D.D., Saito Y., Kuwahara H., Kozuka-Hata H., Shin-I T., Minakuchi Y., Ohishi K., Motoyama A., Aizu T. (2016). Extremotolerant Tardigrade Genome and Improved Radiotolerance of Human Cultured Cells by Tardigrade-Unique Protein. Nat. Commun..

[2] Guidetti R., Rizzo A.M., Altiero T., Rebecchi L. (2012). What Can We Learn from the Toughest Animals of the Earth? Water Bears (Tardigrades) as Multicellular Model Organisms in Order to Perform Scientific Preparations for Lunar Exploration. Planet. Space Sci..

[3] Hengherr S., Worland M.R., Reuner A., Brümmer F., Schill R.O. (2009). High-Temperature Tolerance in Anhydrobiotic Tardigrades Is Limited by Glass Transition. Physiol. Biochem. Zool..

[4] Horikawa D.D., Kunieda T., Abe W., Watanabe M., Nakahara Y., Yukuhiro F., Sakashita T., Hamada N., Wada S., Funayama T. (2008). Establishment of a Rearing System of the Extremotolerant Tardigrade Ramazzottius Varieornatus: A New Model Animal for Astrobiology. Astrobiology.

[5] Ono F., Saigusa M., Uozumi T., Matsushima Y., Ikeda H., Saini N.L., Yamashita M. (2008). Effect of High Hydrostatic Pressure on to Life of the Tiny Animal Tardigrade. J. Phys. Chem. Solids.

[6] Ingemar Jönsson K., Harms-Ringdahl M., Torudd J. (2005). Radiation Tolerance in the Eutardigrade Richtersius Coronifer. Int. J. Radiat. Biol..

[7] Jönsson K.I., Rabbow E., Schill R.O., Harms-Ringdahl M., Rettberg P. (2008). Tardigrades Survive Exposure to Space in Low Earth Orbit. Curr. Biol..

[8] Chavez C., Cruz-Becerra G., Fei J., Kassavetis G.A., Kadonaga J.T. (2019). The Tardigrade Damage Suppressor Protein Binds to Nucleosomes and Protects DNA from Hydroxyl Radicals. eLife.

[9] Dunker A., Lawson J.D., Brown C.J., Williams R.M., Romero P., Oh J.S., Oldfield C.J., Campen A.M., Ratliff C.M., Hipps K.W. (2001). Intrinsically disordered protein. J. Mol. Graph. Model..

[10] Kirke J., Jin X.-L., Zhang X.-H. (2020). Expression of a Tardigrade Dsup Gene Enhances Genome Protection in Plants. Mol. Biotechnol..

[11] Roy S. (2017). Impact of UV Radiation on Genome Stability and Human Health. Adv. Exp. Med. Biol..

[12] Berens P.J.T., Molinier J. (2020). Formation and Recognition of UV-Induced DNA Damage within Genome Complexity. Int. J. Mol. Sci..

[13] Clancy S. (2008). DNA Damage & Repair: Mechanisms for Maintaining DNA Integrity. Nat. Educ..

[14] Doksani Y., Bermejo R., Fiorani S., Haber J.E., Foiani M. (2009). Replicon Dynamics, Dormant Origin Firing, and Terminal Fork Integrity after Double-Strand Break Formation. Cell.

[15] Jazayeri A., Falck J., Lukas C., Bartek J., Smith G.C.M., Lukas J., Jackson S.P. (2006). ATM- and Cell Cycle-Dependent Regulation of ATR in Response to DNA Double-Strand Breaks. Nat. Cell Biol..

[16] Wagh V., Joshi P., Jariyal H., Chauhan N. (2020). ATM and ATR Checkpoint Kinase Pathways: A Concise Review. Adv. Hum. Biol..

[17] Roy R., Chun J., Powell S.N. (2012). BRCA1 and BRCA2: Different Roles in a Common Pathway of Genome Protection. Nat. Rev. Cancer.

[18] Nandi A., Yan L.-J., Jana C.K., Das N. (2019). Role of Catalase in Oxidative Stress- and Age-Associated Degenerative Diseases. Oxidative Med. Cell. Longev..

[19] Fukai T., Ushio-Fukai M. (2011). Superoxide Dismutases: Role in Redox Signaling, Vascular Function, and Diseases. Antioxid. Redox Signal..

[20] Lynch K., Pergolizzi R.G. (2010). In Vitro Method to quantify UV mediated DNA damage. J. Young Investig..

[21] Xiang J., Wan C., Guo R., Guo D. (2016). Is Hydrogen Peroxide a Suitable Apoptosis Inducer for All Cell Types?. Biomed. Res. Int..

[22] Li X., Zhan J., Hou Y., Hou Y., Chen S., Luo D., Luan J., Wang L., Lin D. (2019). Coenzyme Q10 Regulation of Apoptosis and Oxidative Stress in H2O2 Induced BMSC Death by Modulating the Nrf-2/NQO-1 Signaling Pathway and Its Application in a Model of Spinal Cord Injury. Oxid Med. Cell Longev..

[23] Redza-Dutordoir M., Averill-Bates D.A. (2016). Activation of apoptosis signalling pathways by reactive oxygen species. Biochim. Biophys. Acta.

[24] Marinho H.S., Real C., Cyrne L., Soares H., Antunes F. (2014). Hydrogen Peroxide Sensing, Signaling and Regulation of Transcription Factors. Redox Biol..

[25] Pregi N., Belluscio L.M., Berardino B.G., Castillo D.S., Cánepa E.T. (2017). Oxidative Stress-Induced CREB Upregulation Promotes DNA Damage Repair Prior to Neuronal Cell Death Protection. Mol. Cell Biochem..

[26] Shi Y., Venkataraman S.L., Dodson G.E., Mabb A.M., LeBlanc S., Tibbetts R.S. (2004). Direct Regulation of CREB Transcriptional Activity by ATM in Response to Genotoxic Stress. Proc. Natl. Acad. Sci. USA.

[27] Rajabi H.N., Baluchamy S., Kolli S., Nag A., Srinivas R., Raychaudhuri P., Thimmapaya B. (2005). Effects of Depletion of CREB-Binding Protein on c-Myc Regulation and Cell Cycle G1-S Transition. J. Biol. Chem..

[28] Benassi B., Fanciulli M., Fiorentino F., Porrello A., Chiorino G., Loda M., Zupi G., Biroccio A. (2006). C-Myc Phosphorylation Is Required for Cellular Response to Oxidative Stress. Mol. Cell.

[29] Ray Chaudhuri A., Nussenzweig A. (2017). The Multifaceted Roles of PARP1 in DNA Repair and Chromatin Remodelling. Nat. Rev. Mol. Cell Biol..

[30] Ali A.A.E., Timinszky G., Arribas-Bosacoma R., Kozlowski M., Hassa P.O., Hassler M., Ladurner A.G., Pearl L.H., Oliver A.W. (2012). The Zinc-Finger Domains of PARP1 Cooperate to Recognize DNA Strand Breaks. Nat. Struct. Mol. Biol..

[31] Li M., Yu X. (2013). Function of BRCA1 in the DNA Damage Response Is Mediated by ADP-Ribosylation. Cancer Cell.

[32] Indran I.R., Hande M.P., Pervaiz S. (2011). HTERT Overexpression Alleviates Intracellular ROS Production, Improves Mitochondrial Function, and Inhibits ROS-Mediated Apoptosis in Cancer Cells. Cancer Res..

[33] Xiong J., Fan S., Meng Q., Schramm L., Wang C., Bouzahza B., Zhou J., Zafonte B., Goldberg I.D., Haddad B.R. (2003). BRCA1 Inhibition of Telomerase Activity in Cultured Cells. Mol. Cell. Biol..

[34] Dell’Orco M., Milani P., Arrigoni L., Pansarasa O., Sardone V., Maffioli E., Polveraccio F., Bordoni M., Diamanti L., Ceroni M. (2016). Hydrogen Peroxide-Mediated Induction of SOD1 Gene Transcription Is Independent from Nrf2 in a Cellular Model of Neurodegeneration. Biochim. Biophys. Acta (BBA)—Gene Regul. Mech..

[35] Eleutherio E.C.A., Silva Magalhães R.S., de Araújo Brasil A., Monteiro Neto J.R., de Holanda Paranhos L. (2021). SOD1, More than Just an Antioxidant. Arch. Biochem. Biophys..

[36] Glorieux C., Calderon P.B. (2017). Catalase, a Remarkable Enzyme: Targeting the Oldest Antioxidant Enzyme to Find a New Cancer Treatment Approach. Biol. Chem..

[37] Marais T.L.D., Kluz T., Xu D., Zhang X., Gesumaria L., Matsui M.S., Costa M., Sun H. (2017). Transcription Factors and Stress Response Gene Alterations in Human Keratinocytes Following Solar Simulated Ultra Violet Radiation. Sci. Rep..

[38] Ismail A., Yusuf N. (2014). Type I Interferons: Key Players in Normal Skin and Select Cutaneous Malignancies. Dermatol. Res. Pract..

[39] Aragane Y., Kulms D., Luger T.A., Schwarz T. (1997). Down-Regulation of Interferon -Activated STAT1 by UV Light. Proc. Natl. Acad. Sci. USA.

[40] Shaulian E., Schreiber M., Piu F., Beeche M., Wagner E.F., Karin M. (2000). The Mammalian UV Response: C-Jun Induction Is Required for Exit from P53-Imposed Growth Arrest. Cell.

[41] Herold S., Wanzel M., Beuger V., Frohme C., Beul D., Hillukkala T., Syvaoja J., Saluz H.-P., Haenel F., Eilers M. (2002). Negative Regulation of the Mammalian UV Response by Myc through Association with Miz-1. Mol. Cell.

[42] Pathania S., Nguyen J., Hill S.J., Scully R., Adelmant G.O., Marto J.A., Feunteun J., Livingston D.M. (2011). BRCA1 Is Required for Postreplication Repair after UV-Induced DNA Damage. Mol. Cell.

[43] Pavey S., Pinder A., Fernando W., D’Arcy N., Matigian N., Skalamera D., Lê Cao K., Loo-Oey D., Hill M.M., Stark M. (2020). Multiple Interaction Nodes Define the Postreplication Repair Response to UV-induced DNA Damage That Is Defective in Melanomas and Correlated with UV Signature Mutation Load. Mol. Oncol..

[44] Lefkofsky H.B., Veloso A., Ljungman M. (2015). Transcriptional and Post-Transcriptional Regulation of Nucleotide Excision Repair Genes in Human Cells. Mutat. Res. Fundam. Mol. Mech. Mutagen..

[45] Ohashi E., Takeishi Y., Ueda S., Tsurimoto T. (2014). Interaction between Rad9–Hus1–Rad1 and TopBP1 Activates ATR–ATRIP and Promotes TopBP1 Recruitment to Sites of UV-Damage. DNA Repair.

[46] Stepanenko A.A., Heng H.H. (2017). Transient and stable vector transfection: Pitfalls, off-target effects, artifacts. Mutat. Res. Rev. Mutat. Res..

