# Supplementary material for: The Tardigrade Damage Suppressor Protein Modulates Transcription Factor and DNA Repair Genes in Human Cells Treated with Hydroxyl Radicals and UV-C
Source: Biology (Basel). 2021 Sep 27;10(10):970. doi: 10.3390/biology10100970 (PMC8533384; doi:10.3390/biology10100970)
Supplement: Supplementary file 1 [file biology-10-00970-s001.zip › Supplementary tables.pdf]

Supplementary Table S1. Primer sequences

Gene	Primer sequence
ATM	**PF**: 5'-GGCTATTCAGTGTGCGAGAC-3'<br>**PR**: 5'-TTGGGTTTTGGCTCCTTTCG-3'
ATR	**PF**: 5'-ACAGGGGATATTGGAAGGGC-3'<br>**PR**: 5'-TCGGAAGTGCTGTCATCTGA-3'
Bcl2	**PF**: 5'-GCCTTCTTTGAGTTCGGTGG-3'<br>**PR**: 5'-GAAATCAAACAGAGGCCGCA-3'
BRCA1	**PF**: 5'-AGAAAGGGCCTTCACAGTGT-3'<br>**PR**: 5'-TTTGGCACGGTTTCTGTAGC-3'
BRCA2	**PF**: 5'-AGTCTAGGAGCTGAGGTGGA-3'<br>**PR**: 5'-TCCATGACTTGCAGCTTCTC-3'
CASP3	**PF**: 5'-ACTGGACTGTGGCATTGAGA-3'<br>**PR**: 5'- GCACAAAGCGACTGGATGAA-3'
CASP8	**PF**: 5'-GGAGGAGTTGTGTGGGGTAA-3'<br>**PR**: 5'-CCTGCATCCAAGTGTGTTCC-3'
CAT	**PF:** 5'-GAGCCTACGTCCTGAGTCTC-3'<br>**PR:** 5'-ATCCCGGATGCCATAGTCAG-3'
DDB1	**PF:** 5'-GGGTTTCGTGGATGATCAGC-3'<br>**PR:** 5'-CAGATAGTAGAGGGCCCTGC-3'
DFFB	**PF:** 5'-CACAACGTCAGCCAGAACAT-3'<br>**PR:** 5'-CATTGTACTGCATGGACCGG-3'
ERCC1	**PF:** 5'-CAGCATCATTGTGAGCCCTC-3'<br>**PR:** 5'-GAAGTTCTTCCCCAGGCTCT-3'
ERCC6	**PF:** 5'-TGTTAGGTGGCTGTGGGAAT-3'<br>**PR:** 5'-TCCTTCACCCACTGATGCAT-3'
hTERT	**PF:** 5'-AGCCACGTCTCTACCTTGAC-3'<br>**PR:** 5'-GGCACATGAAGCGTAGGAAG-3'
PARP1	**PF:** 5'-TGGAACATCAAGGACGAGCT-3'<br>**PR:** 5'-CATCGCTCTTGAAGACCAGC-3'
PARP2	**PF:** 5'-CTCCCTTGAAGCCAGAGTCA-3'<br>**PR:** 5'-CTGCCTTGATTTGTGCCACT-3'
RAD1	**PF:** 5'-ATTTCCGAGAACATGCCACG-3'<br>**PR:** 5'-GTGCAGTTAAAGTCCCTGGC-3'
RAD17	**PF:** 5'-ATCTCGGACAGTCTCAGTGG-3'<br>**PR:** 5'-ACATCCCTGACAGAGCAACT-3'
RAD50	**PF:** 5'-CTGTCCAACAAGTCAACCAGG-3'<br>**PR:** 5'-ATTCCACAGTCTGCTCCTCC-3'
SOD1	**PF:** 5'-GGAGACTTGGGCAATGTGAC-3'<br>**PR:** 5'-CACAAGCCAAACGACTTCCA-3'
SOD2	**PF: 5'**-TGGCCAAGGGAGATGTTACA-3'<br>**PR:** 5'-ATTGAAACCAAGCCAACCCC-3'
**XRCC6**	**PF:** 5'-GGATTTGATGGAGCCGGAAC-3'<br>**PR:** 5'-GTGGGTCTTCAGCTCCTCTT-3'

Supplementary Table S2: Gene ontology and gene actions

	Gene ontology	Action/pathway (GeneCards https://www.genecards.org/)
ATM	ATM Serine/Threonine Kinase	Cell cycle checkpoint kinase that function as a regulator of a wide variety of downstream proteins, including tumor suppressor proteins p53 and BRCA1, checkpoint kinase CHK2, checkpoint proteins RAD17 and RAD9, and DNA repair protein NBS1. This protein and the closely related kinase ATR are thought to be master controllers of cell cycle checkpoint signaling pathways that are required for cell response to DNA damage and for genome stability.
ATR	ATR Serine/Threonine Kinase	Serine/threonine kinase and DNA damage sensor, activating cell cycle checkpoint signaling upon DNA stress. The encoded protein can phosphorylate and activate several proteins involved in the inhibition of DNA replication and mitosis, and can promote DNA repair, recombination, and apoptosis.
Bcl2	Bcl2 Apoptosis Regulator	Integral outer mitochondrial membrane protein that blocks the apoptotic death of some cells
BRCA1	BRCA1 DNA Repair Associated	DNA repair of double-stranded breaks
BRCA2	BRCA2 DNA Repair Associated	DNA repair of double-stranded breaks
CASP3	Caspase 3	Cysteine-aspartic acid protease that plays a central role in the execution-phase of cell apoptosis. The encoded protein cleaves and inactivates poly(ADP-ribose) polymerase while it cleaves and activates sterol regulatory element binding proteins as well as caspases 6, 7, and 9.
CASP8	Caspase 8	Apoptosis. This protein is involved in the programmed cell death induced by Fas and various apoptotic stimuli.
CAT	Catalase	Converts the reactive oxygen species hydrogen peroxide to water and oxygen and thereby mitigates the toxic effects of hydrogen peroxide.
DDB1	Damage Specific DNA Binding Protein 1	Large subunit (p127) of the heterodimeric DNA damage-binding (DDB) complex
DFFB	DNA Fragmentation Factor Subunit Beta	DFFA is the substrate for caspase-3 and triggers DNA fragmentation during apoptosis
ERCC1	ERCC Excision Repair 1, Endonuclease Non-Catalytic Subunit	Functions in the nucleotide excision repair pathway, and is required for the repair of DNA lesions such as those induced by UV light
ERCC6	ERCC Excision Repair 6, Chromatin Remodeling Factor	Transcription-coupled excision repair. The encoded protein has ATP-stimulated ATPase activity, interacts with several transcription and excision repair proteins, and may promote complex formation at DNA repair sites.
hTERT	Telomerase	Ribonucleoprotein polymerase that maintains telomere ends by addition of the telomere repeat TTAGGG. Telomerase also participates in chromosomal repair
PARP1	Poly [ADP-ribose] polymerase 1	recovery of cell from DNA damage
PARP2	Poly [ADP-ribose] polymerase 2	recovery of cell from DNA damage
RAD1	RAD1 Checkpoint DNA Exonuclease	Stop cell cycle progression in response to DNA damage or incomplete DNA replication
RAD17	RAD17 Checkpoint Clamp Loader Component	Cell cycle checkpoint gene required for cell cycle arrest and DNA damage repair in response to DNA damage
RAD50	RAD50 Double Strand Break Repair Protein	This protein is important for DNA double-strand break repair, cell cycle checkpoint activation, telomere maintenance, and

		meiotic recombination.
SOD1	Superoxide Dismutase 1	The protein encoded by this gene binds copper and zinc ions and is one of two isozymes responsible for destroying free superoxide radicals in the body. The encoded isozyme is a soluble cytoplasmic protein, acting as a homodimer to convert naturally-occuring but harmful superoxide radicals to molecular oxygen and hydrogen peroxide.
SOD2	Superoxide Dismutase 2	It encodes a mitochondrial protein that binds to the superoxide products of oxidative phosphorylation and converts them to hydrogen peroxide and diatomic oxygen.
XRCC6	X-Ray Repair Cross Complementing 6	The p70/p80 autoantigen is a nuclear complex consisting of two subunits with molecular masses of approximately 70 and 80 kDa. The complex functions as a single-stranded DNA-dependent ATP-dependent helicase.
