# Supplementary figures and images for: The Tardigrade Damage Suppressor Protein Modulates Transcription Factor and DNA Repair Genes in Human Cells Treated with Hydroxyl Radicals and UV-C
Source: Biology (Basel). 2021 Sep 27;10(10):970. doi: 10.3390/biology10100970 (PMC8533384; doi:10.3390/biology10100970)

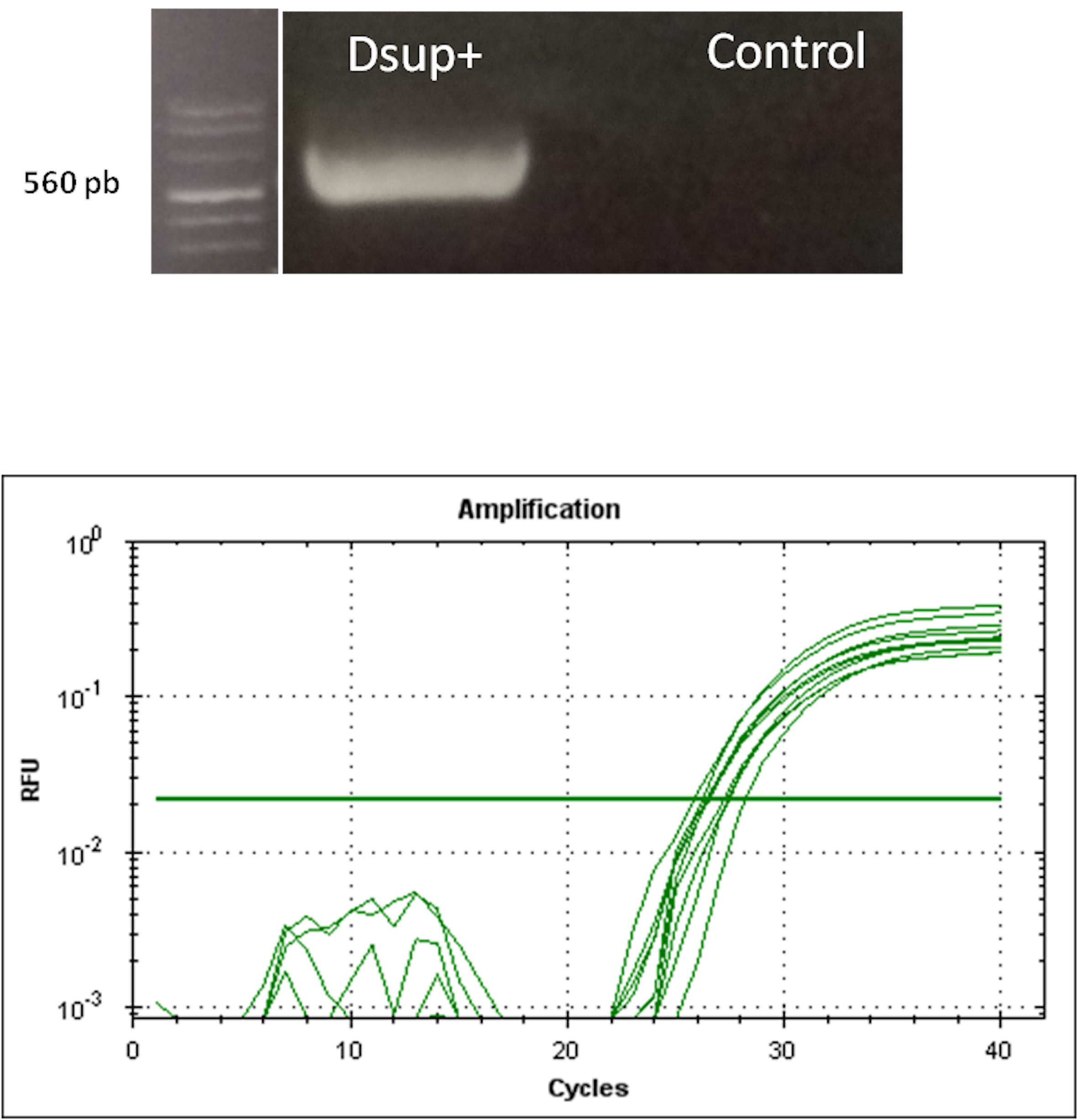

Supplement: Supplementary file 1 [file biology-10-00970-s001.zip › Supplementary Figures/SupplFigS1.jpg]

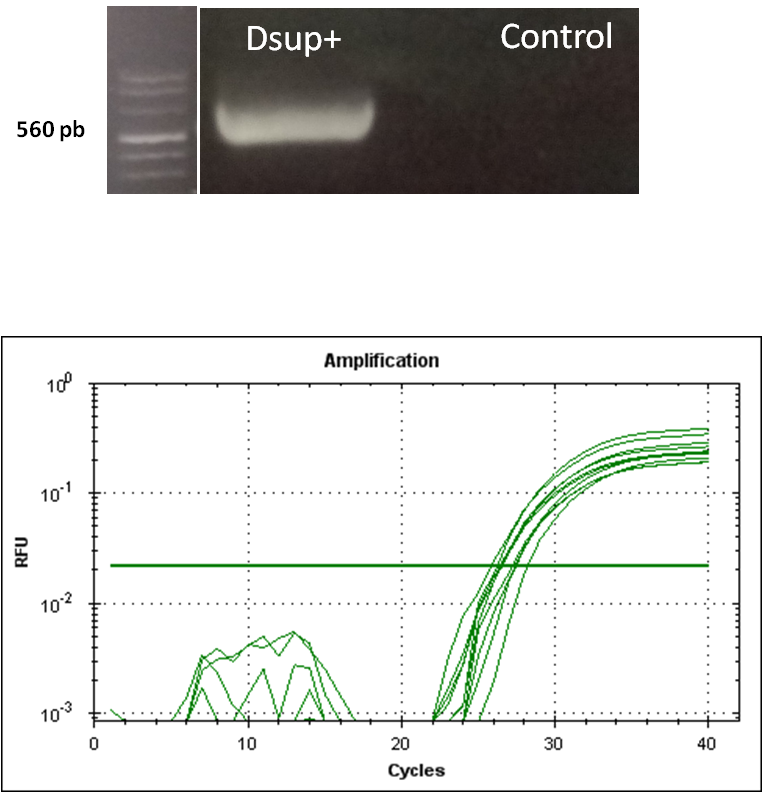

Supplement: Supplementary file 1 [file biology-10-00970-s001.zip › Supplementary Figures/SupplFigS1.png]

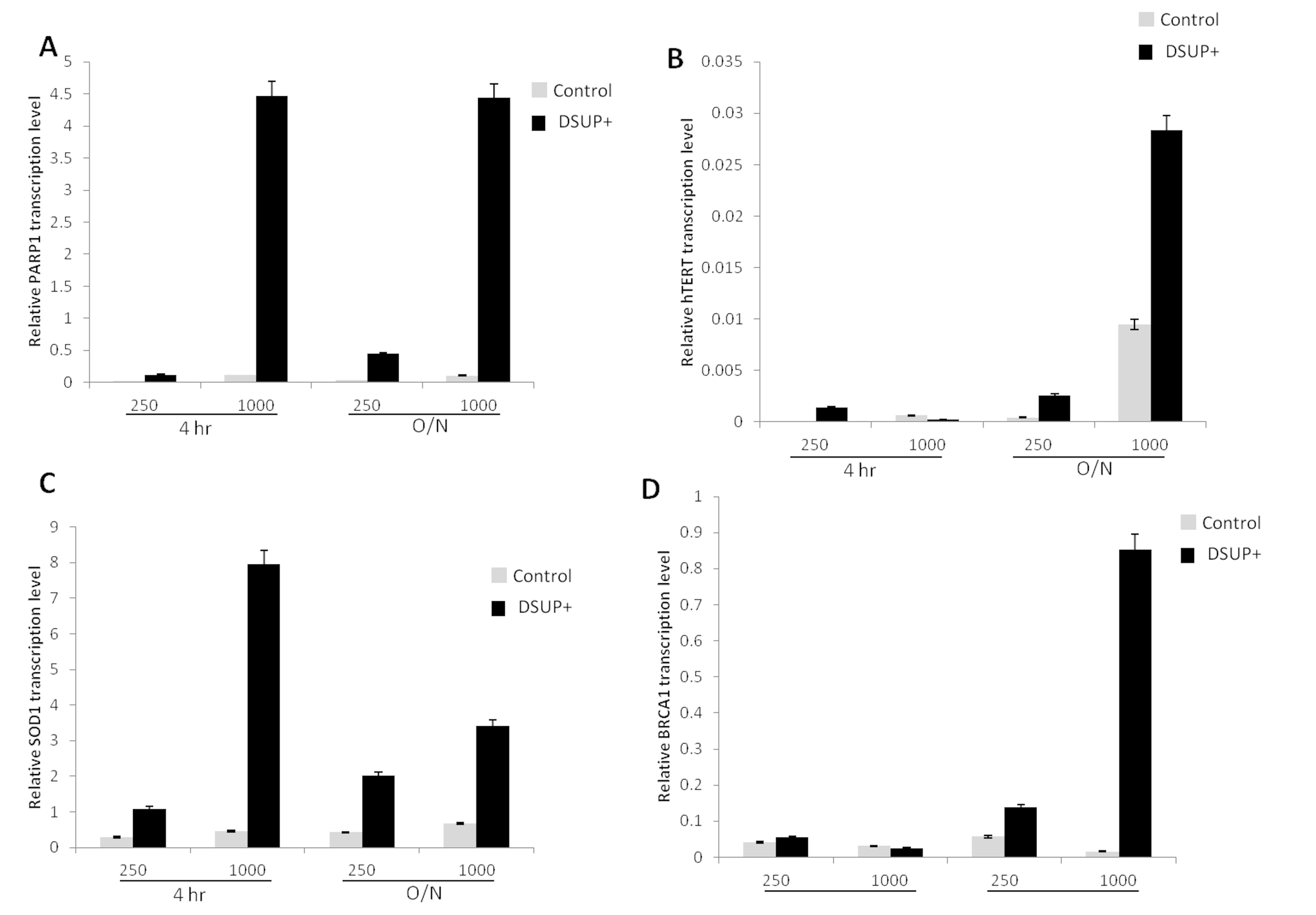

Supplement: Supplementary file 1 [file biology-10-00970-s001.zip › Supplementary Figures/SupplFigS2.jpg]

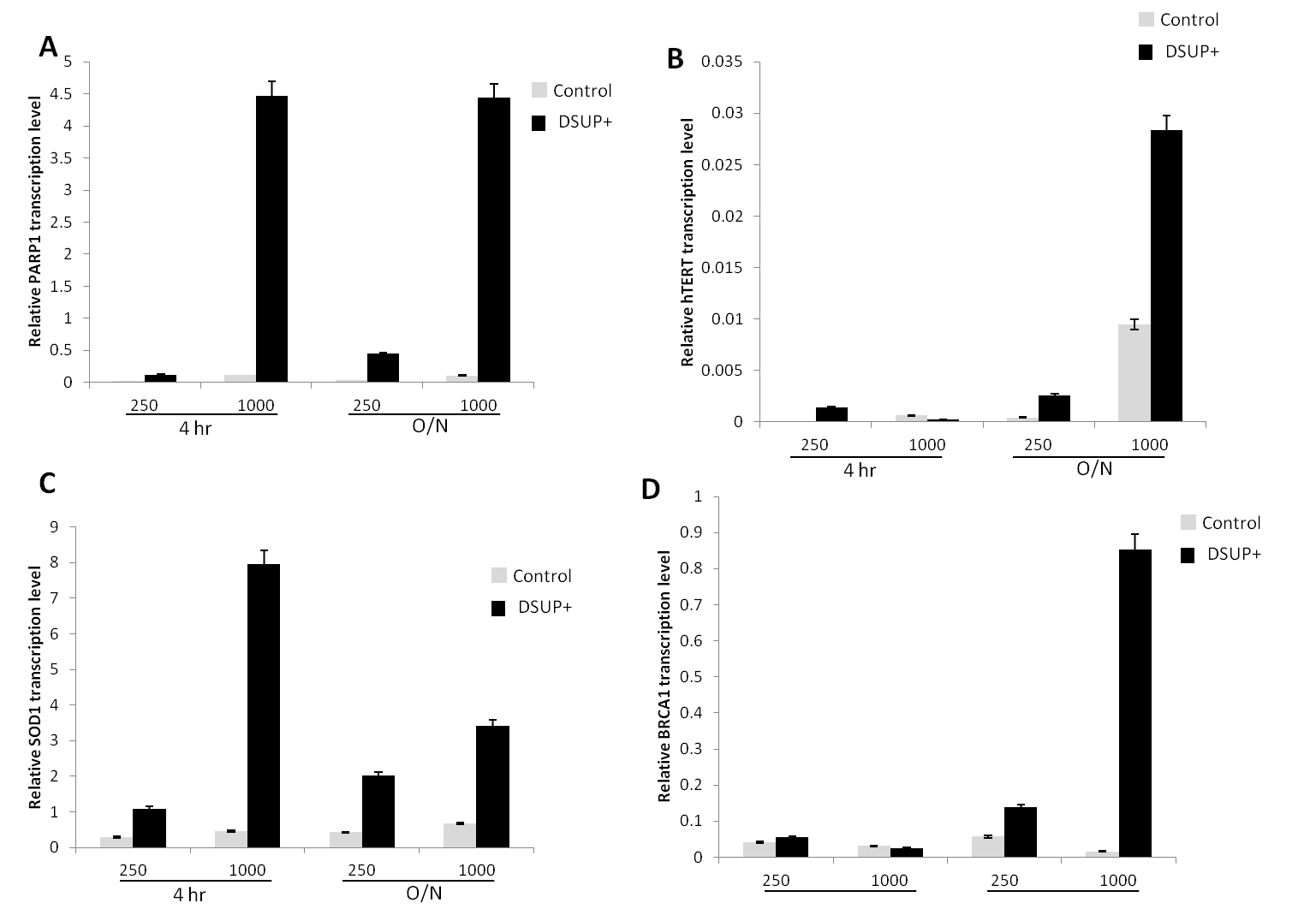

Supplement: Supplementary file 1 [file biology-10-00970-s001.zip › Supplementary Figures/SupplFigS2.png]

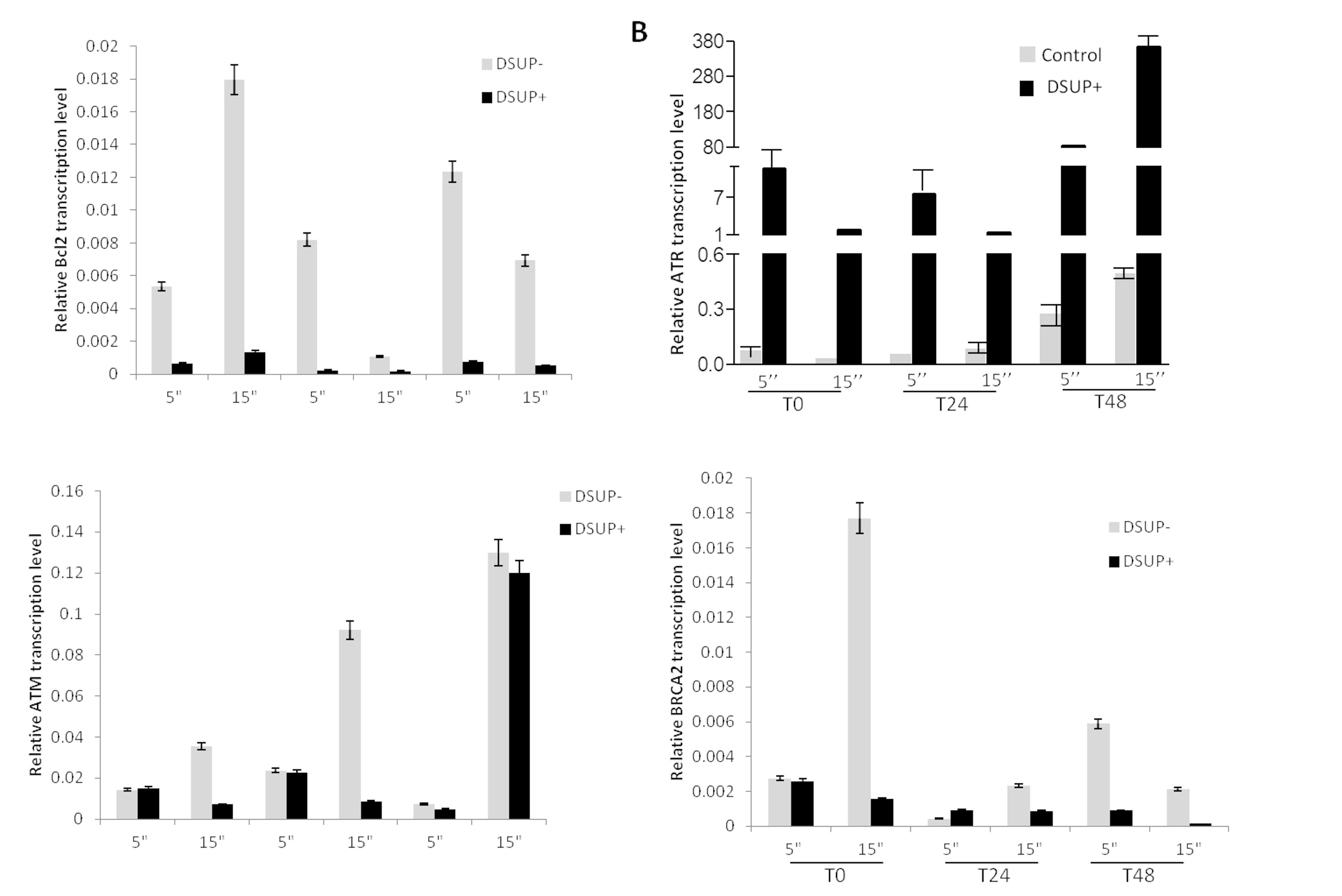

Supplement: Supplementary file 1 [file biology-10-00970-s001.zip › Supplementary Figures/SupplFigS3.jpg]

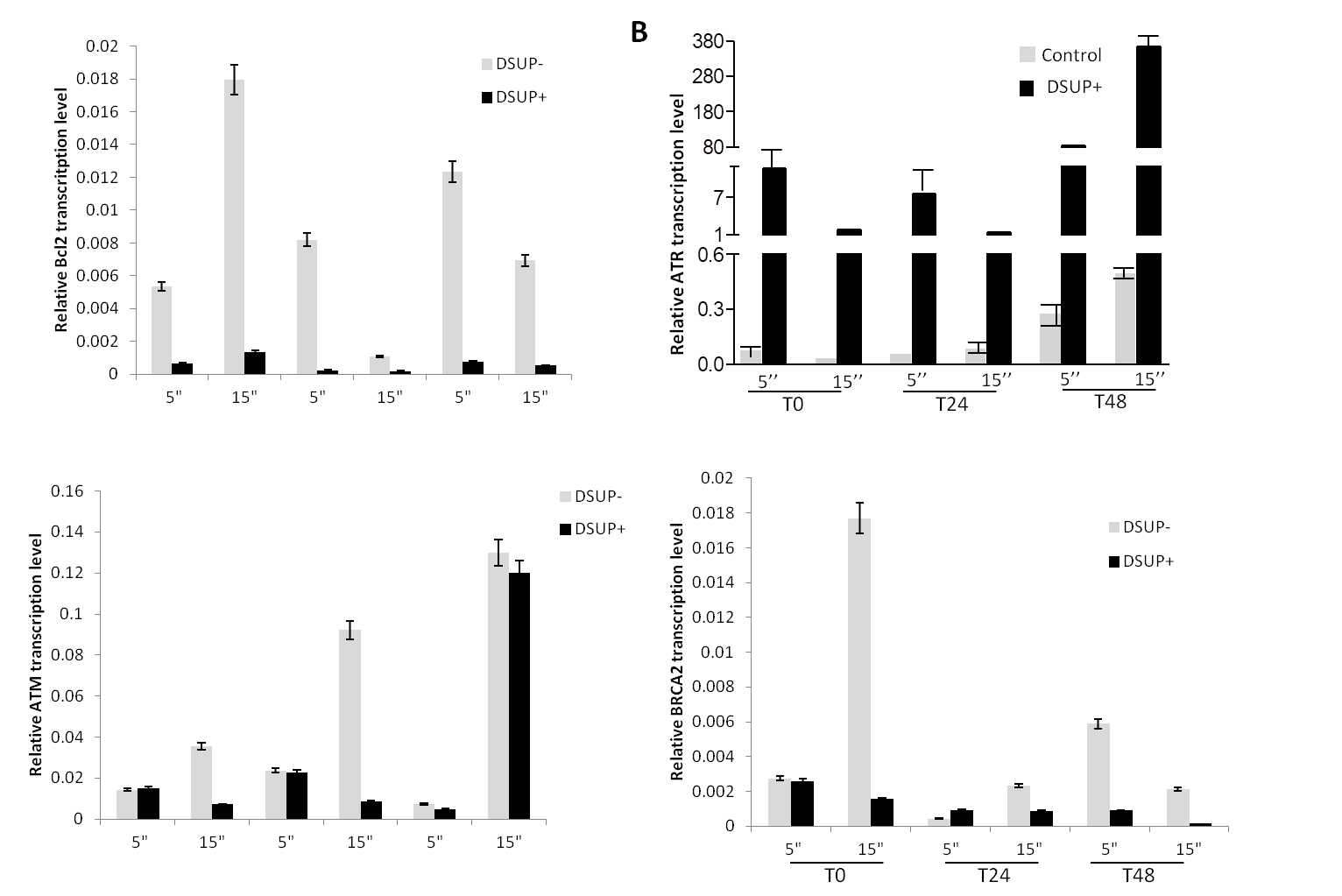

Supplement: Supplementary file 1 [file biology-10-00970-s001.zip › Supplementary Figures/SupplFigS3.png]
